# Geriatric nutritional risk index has a prognostic value for recovery outcomes in elderly patients with brain abscess

**DOI:** 10.3389/fnut.2024.1410483

**Published:** 2024-07-18

**Authors:** Xu Pei, Yutu Zhang, Dongfeng Jiang, Meng Zhang, Junyan Fu, Yang Niu, Mi Tian, Shanshan Huang

**Affiliations:** ^1^Department of Critical Care Medicine and Neurosurgery of Huashan Hospital, State Key Laboratory of Medical Neurobiology and MOE Frontiers Center for Brain Science, Institutes of Brain Science, Fudan University, Shanghai, China; ^2^Department of General Practice of Huashan Hospital, Fudan University, Shanghai, China; ^3^Department of Infectious Diseases of Huashan Hospital, Fudan University, Shanghai, China; ^4^Department of Neurosurgery, Liaocheng People's Hospital, Liaocheng, China; ^5^Department of Radiology of Huashan Hospital, Fudan University, Shanghai, China; ^6^Department of Clinical Nutrition, Xinhua Hospital Affiliated to Shanghai Jiaotong University School of Medicine, Shanghai, China; ^7^Department of Geriatric of Huashan Hospital, National Clinical Research Center for Aging and Medicine, Fudan University, Shanghai, China

**Keywords:** geriatric nutritional risk index, malnutrition, brain abscess, recovery outcomes, prognosis

## Abstract

**Background:**

The Geriatric Nutritional Risk Index (GNRI) is a straightforward and objective tool for nutritional screening in older patients and has been demonstrated to possess prognostic predictive value in several diseases. Nonetheless, there is a lack of research on the nutritional risk associated with brain abscess in the older. This study aimed to evaluate the prevalence of nutritional risk among these patients by GNRI and to investigate its potential prognostic value for clinical outcomes.

**Materials and methods:**

From August 2019 to April 2023, 100 older patients diagnosed with brain abscess were enrolled in this single-center prospective cohort study, which evaluated the prognostic value of the Geriatric Nutritional Risk Index (GNRI) in elderly brain abscess patients. Data collected included demographic, and clinical characteristics at admission and calculated the GNRI, and the Glasgow Outcome Scale (GOS) score 6 months post-discharge. A GOS score of 5 was considered indicative of a good recovery, whereas scores ranging from 1 to 4 were classified as poor recovery.

**Results:**

The results revealed that 48% of older brain abscess patients were at risk of malnutrition according to the GNRI. These patients had significantly higher post-admission C-reactive protein (CRP) levels (*p* = 0.017), more comorbidities (*p* < 0.001), and higher age-adjusted Charlson Comorbidity Index (aCCI) scores (*p* < 0.001) compared to those without nutritional risk. Spearman correlation analysis showed that GNRI scores were negatively correlated with CRP levels, comorbidities, and aCCI scores, and positively correlated with Glasgow Outcome Scale (GOS) scores (Spearman’s ρ = 0.624, *p* < 0.001). Multivariate logistic regression revealed that lower GNRI values were linked to reduced GOS levels (OR = 0.826, 95% CI: 0.775–0.880). ROC analysis determined a GNRI threshold of 97.50 for predicting poor recovery, with 90.57% sensitivity and 87.23% specificity.

**Conclusion:**

The older brain abscess patients exhibited a high malnutrition risk. GNRI showed an important predictive value for recovery in older patients, which could be helpful in clinical intervention and rehabilitation.

## Introduction

Brain abscess, a serious intracranial infection, manifests as a pus collection within the brain tissue (1). Its complex pathogenesis involves a variety of infectious agents, such as bacteria (2), fungi (3), and parasites (4). Risk factors for the formation of a brain abscess include skull injuries, peripheral infections, a compromised immune system, malnutrition, and factors associated with advanced age. Brain abscesses can lead to severe consequences such as increased intracranial pressure, consciousness disturbances, brain herniation, abscess rupture causing meningitis or ventriculitis, focal neurological deficits, and seizures (5). The mortality rate for brain abscesses is around 10%, with 45% of affected patients experiencing neurological deficits post-treatment (4, 6, 7). In older patients, this mortality rate increases, with studies indicating a range of 20 to 40% (4, 7). The Glasgow outcome scale (GOS) is a scale used to systematically evaluate post-brain injury recovery, categorizing outcomes into five levels: death, vegetative state, severe disability, moderate disability, and good recovery (8). The GOS has been employed widely as a metric to assess the effectiveness of brain injury treatment and rehabilitation strategies (9, 10).

Nutritional status significantly influences the progression of brain abscesses and the efficacy of treatments (11). Inadequate nutrition weakens immune defenses, increasing infection risks (12). Furthermore, nutritional health is vital during treatment and recovery, with nutritional deficits potentially delaying wound healing, impeding neurological recovery, and prolonging the recovery process (13). Such deficiencies can negatively impact patient lifespan, and quality of life, and impose significant economic costs (14). The incidence of malnutrition and risk of nutritional deficits in elderly populations within hospital and nursing facility settings is nearly 50%. This condition is detrimental, with a profound link to clinical outcomes, and it significantly amplifies the risk of geriatric syndromes including dependency in activities of daily living, myasthenia gravis, and frailty (15). Given the heightened risk of malnutrition among the older due to aging, conducting a simple and objective assessment of malnutrition risk is imperative to develop effective nutritional interventions. The geriatric nutritional risk index (GNRI) serves as an objective screen tool to evaluate the nutritional status of older individuals and its association with health risks (16). The GNRI has emerged as a powerful prognostic indicator for the older, particularly regarding long-term postoperative outcomes (17–19). Numerous studies have demonstrated the utility of the GNRI in predicting outcomes among a wide range of clinical conditions in the older, including chronic kidney disease (20), heart failure (21), chronic obstructive pulmonary disease (22), and sepsis (23). Additionally, GNRIs have relevant applications in brain-related injuries. Su et al.’s study revealed that the GNRI is a prognostic factor for death in older patients with moderate to severe traumatic brain injuries (24). Furthermore, evidence from another study highlighted that a lower GNRI is an independent predictor of brain infarction and hemorrhage in patients on maintenance hemodialysis (25). Similarly, the study by Dai et al. demonstrated that GNRI was related to the risk of stroke-associated pneumonia (26).

To our knowledge, no study has examined the nutritional status of brain abscess patients and its correlation with clinical outcomes. Therefore, this study employed the GNRI to assess the risk of malnutrition among older brain abscess patients, exploring the relationship between nutritional status and clinical features. Additionally, we employed the GOS to evaluate recovery post-discharge in these patients, further exploring GNRI’s predictive value for GOS scores in older brain abscess patients’ post-discharge.

## Materials and methods

### Study design and participants

**Figure 1:**
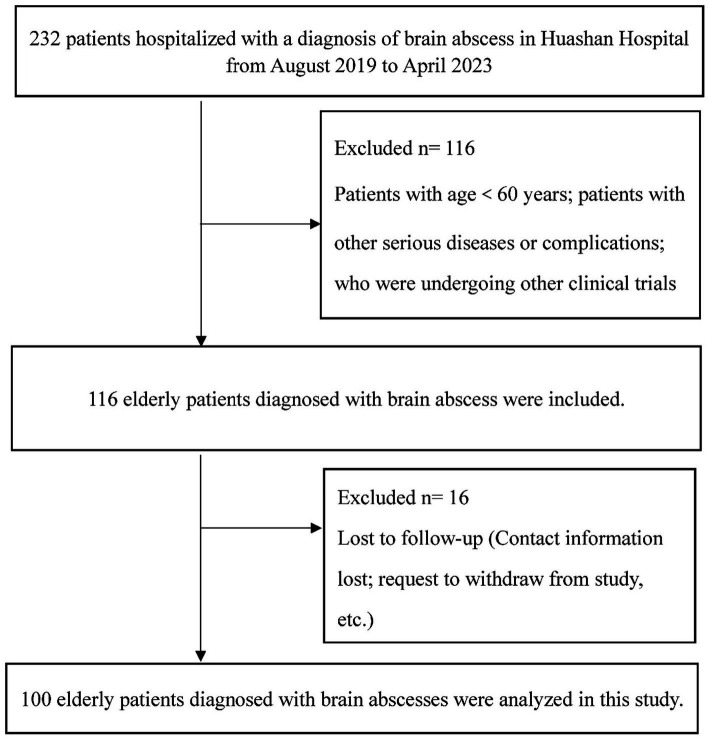
Flow chart of included patients with brain abscess in the current analysis.

This single-center prospective cohort study was executed at Huashan Hospital, Fudan University. The study population comprised individuals admitted with a diagnosis of brain abscess between August 2019 and April 2023. Diagnosis of brain abscess in surgical patients was confirmed through pathology and pus culture, while conservatively managed patients were diagnosed through cerebrospinal fluid culture or in conjunction with magnetic resonance imaging and clinical manifestations (27). Patients were subjected to a follow-up period of 6 months post-discharge. The inclusion criteria for this study were as follows: (a) patients diagnosed with brain abscess confirmed by imaging and/or cerebrospinal fluid culture; (b) participants must be aged 60 or older; (c) voluntary participation and signed informed consent. The exclusion criteria for this study encompass the following: (a) individuals under the age of 60; (b) patients who are lost to follow-up; (c) those with severe health conditions such as heart or lung failure, advanced cancer, or other critical illnesses that may influence nutritional status and the assessment of prognosis; (d) participants who are unconscious or have a mental illness that prevents them from cooperating with the assessment process (Figure 1). Ethical endorsement for this study’s protocol was granted by the Research Ethics Committee of Huashan Hospital, Fudan University. This approval was secured to ascertain adherence to the ethical guidelines outlined in the Declaration of Helsinki (1975), as well as its later amendments, thereby ensuring the protection of participants’ rights and well-being throughout the research.

### Data collection

#### Demographic and clinical characteristics

Demographic and clinical data were systematically gathered, encompassing age, gender, and histories of tobacco use and alcohol consumption, which were obtained through patient history collection. Serum albumin levels and C-reactive protein (CRP) concentrations were documented for all subjects upon their initial evaluation, conducted within the first 48 h post-admission. These blood indicators were collected through routine blood tests (Sysmex XN-9000 analyzer) and biochemical analyses (VITROS 5600 Integrated System) performed by the laboratory department, ensuring accurate and standardized measurements. Additional details regarding therapeutic interventions employed, comorbidities were also collected. Comorbid conditions encompass a spectrum of diseases and disorders, including chronic illnesses (e.g., hypertension, diabetes mellitus, cardiovascular disorders, pulmonary pathologies, renal dysfunctions), psychiatric disorders (e.g., depressive disorders, anxiety disorders, bipolar affective disorders), autoimmune diseases (e.g., rheumatoid arthritis, systemic lupus erythematosus), neurological pathologies (e.g., Alzheimer’s disease, Parkinson’s disease), metabolic disorders (e.g., obesity, dyslipidemia), infectious diseases (e.g., chronic hepatitis, HIV/AIDS), and gastroenterological conditions (e.g., chronic gastritis, inflammatory bowel disease). The age-adjusted Charlson Comorbidity Index (aCCI) serves as a pivotal tool for assessing mortality risks linked to a spectrum of prognostic clinical indicators. We utilized this index to quantify the cumulative risk profile of patient populations (28).

#### Nutritional assessment

Anthropometric parameters were ascertained by trained medical personnel employing a calibrated scale equipped with a stadiometer (RGZ 120, China). Body mass index (BMI) was calculated utilizing the formula: weight (kg) /height2 (m^2^). The classification of BMI adhered to the guidelines established by the Chinese Obesity Working Group, delineating the categories as follows: underweight (<18.5 kg/m^2^), normal weight (18.5–23.9 kg/m^2^), and overweight (>23.9 kg/m^2^).

The GNRI is derived through the following equation (16):


$$\begin{array}{l} \mathrm{GNRI}=\left(14.89^{*}\mathrm{albumin}\left(g/L\right)\right)/22.0+\left(41.7^{*}\mathrm{weight}\left(\mathrm{kg}\right)\right)/\\ \left(\mathrm{ideal weight}\left(\mathrm{kg}\right)\right)\text{.} \end{array}$$

The ideal weight within this equation is ascertained via the Lorenz formula, which is contingent upon the individual’s sex and stature. Should the ratio of the current to ideal weight exceed 1, it is subsequently adjusted to 1.

Lorenz formula:


$$\mathrm{For male}:\mathrm{ideal weight}\;\left(\mathrm{kg}\right)=\mathrm{height}\left(\mathrm{cm}\right)-100-\left(\begin{array}{l} \mathrm{height}\\ \left(\mathrm{cm}\right)-150 \end{array}\right)/4.$$

$$\mathrm{For female}:\mathrm{ideal weight}\;\left(\mathrm{kg}\right)=\mathrm{height}\left(\mathrm{cm}\right)-100-\left(\begin{array}{l} \mathrm{height}\\ \left(\mathrm{cm}\right)-\\ 150 \end{array}\right)/2.5\text{.}$$

#### Outcomes

The length of stay (LOS) of each patient was recorded. LOS was defined as the interval, measured in days, extending from the initial date of admission to the terminal date of discharge. GOS was employed for the assessment of overall functional recuperation in overall. The scale is stratified into quintuple gradations as follows: GOS = 1, death; GOS = 2, vegetative state; GOS = 3, severe disability; GOS = 4, moderate disability; and GOS = 5, good recovery.

#### Statistical analysis

Participant characteristics in this study were summarized as mean ± standard deviation (SD) for normally distributed continuous variables and median (interquartile range, IQR) for non-normally distributed continuous variables. Categorical variables were reported as frequencies and percentages. The t-test and Wilcoxon rank-sum test assessed differences in key indicators between groups with and without nutritional risk. Pearson correlation analysis explored factors associated with GNRI scores. Univariate logistic regression on GOS included variables with *p* < 0.20 in a subsequent multivariate analysis. Odds ratios (OR) and 95% confidence intervals (CI) were reported. A stratified histogram depicted GOS score distributions across nutritional risk categories in brain abscess patients. The prognostic value of GNRI for the older brain abscess patients was determined using receiver operating characteristic curve (ROC) analysis, a GOS score of 5 was classified as indicative of good recovery, whereas scores ranging from 1 to 4 were considered to reflect poor recovery. Statistical analyses were two-sided, with an alpha level of 0.05, performed using SAS V.9.4 (SAS Institute, Cary, NC, United States). R version 4.3.2, with ggplot2 and pROC packages, was used for histogram and ROC curve visualizations.

## Results

This study’s final analysis included a cohort of 100 older patients diagnosed with brain abscess. Table 1 revealed the median age to be 68 years (IQR 64.5–72), with females constituting 61% (61/100) of the sample. In this cohort, 11% (11/100) were underweight, and 30% (30/100) were overweight. The proportion of alcohol consumption and smoking was noted in 19% (19/100) and 18% (18/100) of the patients, respectively. Comorbidities were prevalent in older brain abscess patients, with the median number of comorbidities being 2 (IQR 0–3). The most common comorbidity was hypertension (36%, *n* = 36), followed by diabetes (31%, *n* = 31). GNRI assessments conducted within 48 h of admission indicated malnutrition risks in 48% of patients, with 4% facing major malnutrition risk, 23% moderate risk, and 21% low risk. The median hospital stay duration was 15 days (IQR 10–22), and the median GOS score at 6 months post-discharge was 5 (IQR 3–5), reflecting generally favorable outcomes.

**Table 1:** Characteristics of this study participants.

	Whole study group (*n* = 100)
Age (year) (median, IQR)	68.0 (64.5–72.0)
Male (*n*, %)	61 (61.00%)
Height (cm) (mean ± SD)	166.05 ± 7.13
Weight (kg) (mean ± SD)	62.22 ± 10.50
BMI (kg/m^2^) (mean ± SD)	22.50 ± 3.15
Underweight (<18.5 kg/m^2^) (*n*, %)	11 (11.0%)
Normal weight (18.5–23.9 kg/m^2^) (*n*, %)	59 (59.0%)
Overweight (>23.9 kg/m^2^) (*n*, %)	30 (30.0%)
Smoking status (*n*, %)	
Current or former	19 (19.0%)
Never	81 (81.0%)
Drinking status (*n*, %)	
Current or former	18 (18.0%)
Never	82 (82.0%)
Comorbidity (median, IQR)	2 (0–3)
aCCI score (median, IQR)	1 (0–2)
LOS (days) (median, IQR)	15 (10–22)
GOS score (median, IQR)	5 (3–5)
Malnutrition risk to GNRI (*n*, %)	48 (48.0%)
Major malnutritional risk (GNRI: <82)	4 (4.0%)
Moderate malnutritional risk (GNRI: 82–91)	23 (23.0%)
Low malnutritional risk (GNRI: 92–98)	21 (21.0%)

IQR, Interquartile range; BMI, Body mass index; aCCI, age-adjusted Charlson Comorbidity Index; LOS, Length of stay; GOS, Glasgow outcome scale; GNRI, Geriatric nutritional risk index.

As shown in Table 2, individuals at risk for malnutrition had significantly lower BMI compared to those not at risk (*p* < 0.001). This group also had a higher median comorbidity count of 3 (IQR 1–4) (*p* < 0.001), suggesting a higher burden of comorbid conditions. Furthermore, aCCI scores were significantly higher in the malnutrition risk group (*p* < 0.001). Admission CRP levels were also higher in the malnutrition risk group than in the non-risk group (*p* = 0.017), indicating more severe inflammation. Treatment strategies varied, with a larger fraction of the malnutrition risk group receiving conservative medical treatment (66.7%) compared to the non-risk group (38.5%) (*p* = 0.003). Additionally, the malnutrition risk group had lower median GOS scores of 3 (IQR 3–4) (*p* < 0.001), indicating a worse prognosis.

**Table 2:** A comparative analysis of demographic and clinical characteristics between patients with and without malnutrition risk according to GNRI.

	No malnutrition risk (*n* = 52)	Malnutrition risk (*n* = 48)	*p* value
Age (year) (mean ± SD)	68.04 ± 5.40	68.77 ± 5.19	0.492
Male (*n*, %)	35 (67.3%)	26 (54.2%)	0.182
BMI (kg/m^2^) (mean ± SD)	24.13 ± 2.83	20.74 ± 2.48	<0.001
Underweight (<18.5 kg/m^2^) (*n*, %)	2 (3.8%)	9 (18.8%)	
Normal weight (18.5–23.9 kg/m^2^) (*n*, %)	25 (48.1%)	34 (70.8%)	
Overweight (>23.9 kg/m^2^) (*n*, %)	25 (48.1%)	5 (10.4%)	
Smoking status (*n*, %)			0.342
Current or former	8 (15.4%)	11 (22.9%)	
Never	44 (84.6%)	37 (77.1%)	
Drinking status (*n*, %)			0.484
Current or former	8 (15.4%)	10 (20.8%)	
Never	44 (84.6%)	38 (79.2%)	
Comorbidity (median, IQR)	1 (0–2)	3 (1–4)	<0.001
aCCI score (median, IQR)	3 (2–3)	3 (3–4)	<0.001
CRP (mg/L) (median, IQR)	4.3 (1.8–16.0)	15.6 (4.9–42.2)	0.017
Treatment modality (*n*, %)			0.003
Conservative therapeutic	20 (38.5%)	32 (66.7%)	
Aspiration	17 (32.7%)	11 (22.9%)	
Excession	15 (28.8%)	5 (10.4%)	
LOS (days) (median, IQR)	14.5(10.0–62.0)	15.5 (9.0–23.5)	0.822
GOS score (median, IQR)	5 (5–5)	3 (3–4)	<0.001

GNRI, Geriatric nutritional risk index; BMI, Body mass index; IQR, Interquartile range; aCCI, age-adjusted Charlson Comorbidity Index; CRP, C-reactive protein; LOS, Length of stay; GOS, Glasgow outcome scale.

**Figure 2:**
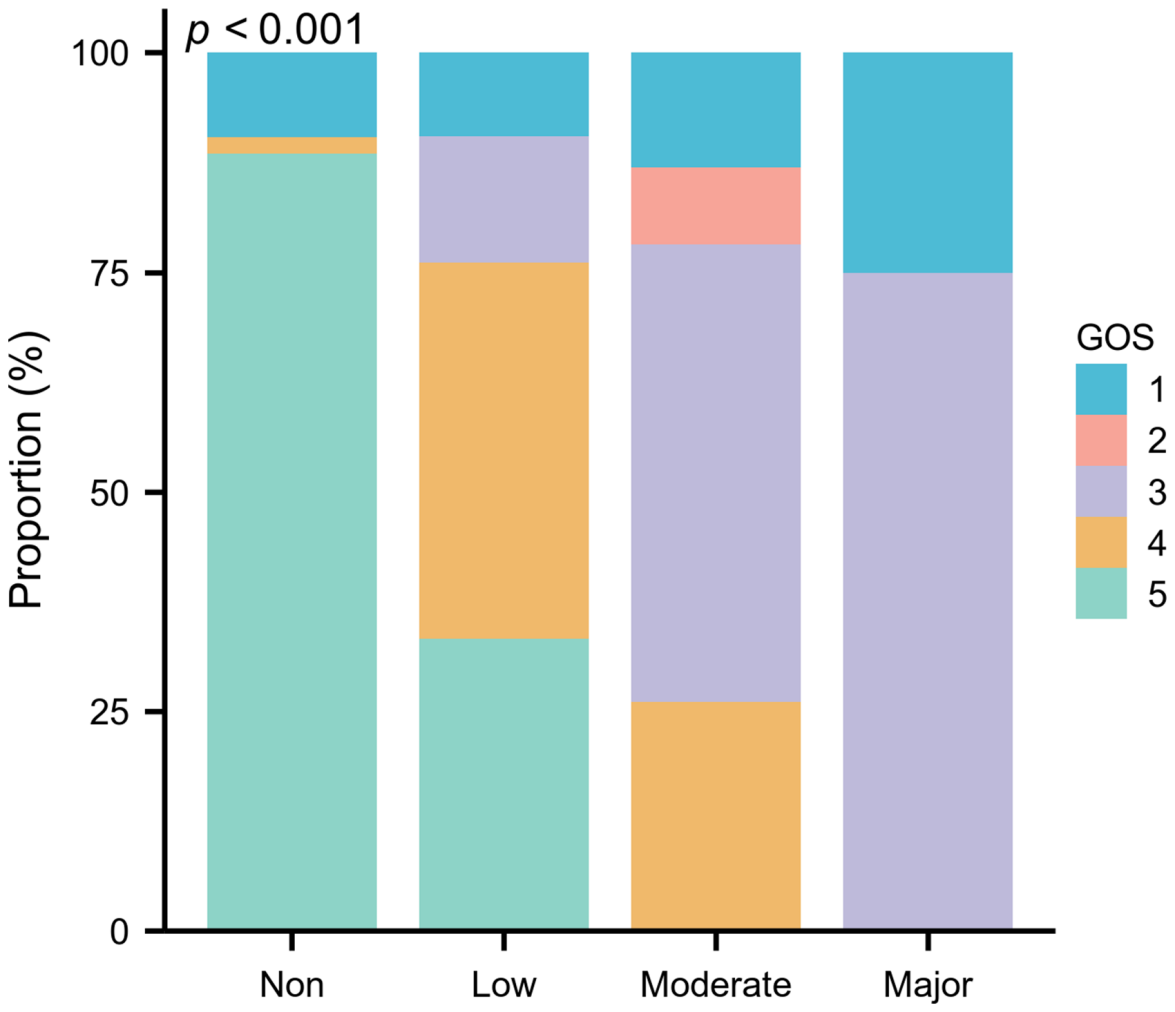
The proportion of GOS scores in different nutritional categories assessed by GNRI among 100 elderly patients with brain abscess.

Table 3 demonstrated the relationships between GNRI scores and various clinical parameters. Specifically, CRP levels (Spearman’s ρ = −0.291, *p* = 0.006), the total number of comorbidities (Spearman’s ρ = −0.284, *p* = 0.004), and aCCI scores (Spearman’s ρ = −0.310, *p* = 0.002) are inversely correlated with GNRI scores. In contrast, GOS scores show a strong positive correlation with GNRI scores (Spearman’s ρ = 0.624, *p* < 0.001), indicating that higher nutritional status is associated with better neurological outcomes. Figure 2 delineates the distribution of GOS scores across varying malnutrition risk categories, with the precise value distribution elaborated in Supplementary Table S1 (*p* < 0.001). Furthermore, by assigning numerical values to surgical methods—1 for conservative therapeutic, 2 for aspiration, and 3 for excision—a positive correlation is observed between these values and GNRI scores (Spearman’s ρ = 0.340, *p* < 0.001). This suggests a preference for conservative treatment in patients with poorer nutritional status, consistent with the trends shown in Table 2.

**Table 3:** Correlation analysis of potential factors associated with GNRI score.

	Spearman’s ρ	*p* value
Age (year)	−0.099	0.328
CRP (mg/L)	−0.291	0.006
Smoking status	−0.182	0.069
Drinking status	−0.139	0.168
Treatment modality	0.340	<0.001
Comorbidity	−0.284	0.004
aCCI score	−0.310	0.002
LOS (days)	−0.020	0.845
GOS score	0.624	<0.001

GNRI, Geriatric nutritional risk index; BMI, Body mass index; aCCI, age-adjusted Charlson Comorbidity Index; CRP, C-reactive protein; LOS, Length of stay; GOS, Glasgow outcome scale.

**Figure 3:**
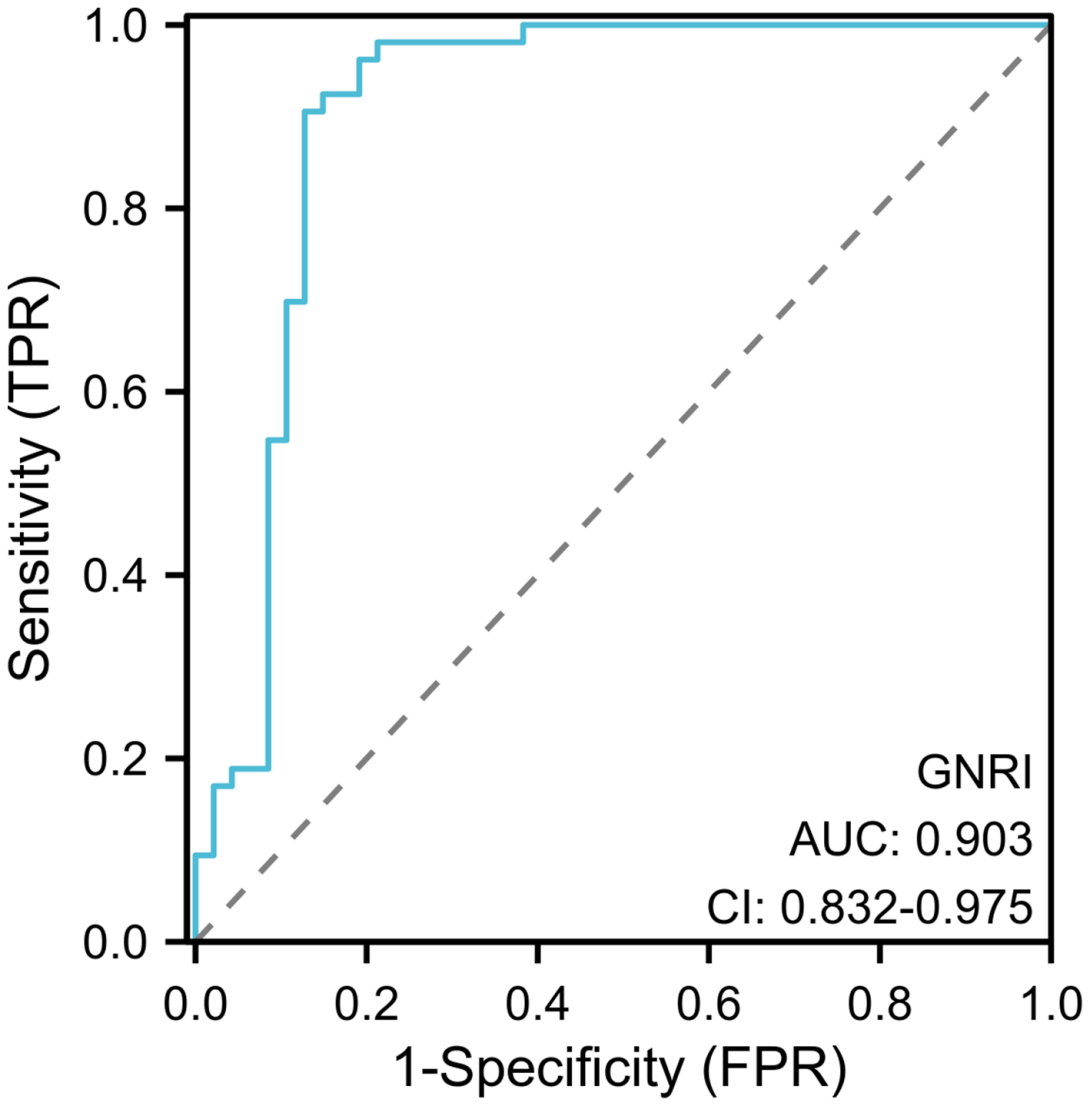
ROC curve analysis of GNRI for poor recovery in the elderly hospitalized with brain abscess.

In the univariate logistic regression analyses, the association of each predictor variable with the GOS score was assessed. The results revealed no significant correlation for age or gender with the GOS score. BMI and GNRI score were significantly negatively correlated with the GOS score, whereas CRP, aCCI score, and number of comorbidities showed a positive correlation with the GOS score. In the subsequent multivariable logistic regression analysis, which incorporated variables with a *p*-value of less than 0.2 from the univariate analysis, GNRI was found to have a significant negative correlation with GOS score (Table 4). This highlights GNRI’s potential as a predictive factor for outcomes. Additionally, Figure 3 showed the ROC curve for GNRI in predicting poor recovery. The analysis yielded an Area Under the Curve (AUC) of 0.903, with the 95% Confidence Interval (CI) extending from 0.832 to 0.975. At the optimal threshold of 97.50, with a sensitivity of 90.57% and a specificity of 87.23% in predicting recovery outcomes.

**Table 4:** Logistic regression analysis for GOS as a dependent variable.

	Univariate		Multivariate	
	OR (95%CI)	*p* value	OR (95%CI)	*p* value
Age	1.063 (0.991–1.141)	0.090	1.012 (0.902–1.135)	0.845
sex	0.520 (0.244–1.109)	0.091	0.829 (0.288–2.384)	0.728
BMI	0.757 (0.656–0.874)	<0.001	0.908 (0.721–1.144)	0.413
Smoking status	1.581 (0.629–3.972)	0.330		
Drinking status	2.413 (0.948–6.146)	0.089	1.154 (0.253–5.265)	0.853
CRP (mg/L)	1.012 (1.004–1.021)	0.003	1.010 (0.999–1.021)	0.073
Treatment modality	0.669 (0.409–1.094)	0.109	1.338 (0.654–2.738)	0.425
Comorbidity	1.216 (1.041–1.420)	0.014	0.969 (0.748–1.254)	0.811
aCCI score	2.024 (1.411–2.903)	<0.001	1.456 (0.800–0.916)	0.219
LOS	0.995 (0.965–1.023)	0.662		
GNRI	0.826 (0.775–0.880)	<0.001	0.830 (0.752–0.916)	<0.001

GOS, Glasgow outcome scale; BMI, Body mass index; CRP, C-reactive protein; aCCI, age-adjusted Charlson Comorbidity Index; LOS, Length of stay; GNRI, Geriatric nutritional risk index.

Univariate: Univariate logistic regression analysis.

Multivariate: Multivariate logistic regression analysis: inclusion of predictors exhibiting univariate logistic regression outcomes with *p* < 0.20.

## Discussion

This study represents the first study into the association between malnutrition risk, as determined by the GNRI, and clinical outcomes in older patients with brain abscess. Findings from this prospective cohort study revealed that nearly half of the older brain abscess patients exhibited malnutritional risk, which is related to their treatment modality as well. Concurrently, the GNRI displayed associations with both the quantum of comorbidities and the aCCI score, underscoring the intricate interplay between nutritional status and the aggregate health burden in this demographic. Notably, older brain abscess patients presenting with lower GNRI scores were observed to have diminished GOS scores, indicating that GNRI has a predictive impact on their clinical outcomes.

Malnutrition, often underreported and underdiagnosed, is widespread among older hospitalized patients, contributing to adverse clinical outcomes such as prolonged hospital stays and elevated mortality rates (29). The GNRI provides an efficient, objective, and age-specific approach to nutritional screening in the older (16). It assesses the nutritional risks of the older in a timely and accurate manner by utilizing current weight metrics and serum albumin levels, thereby minimizing recall bias associated with past weight changes (16). In this study, the GNRI was employed to assess the malnutrition risk in older patients with brain abscesses, revealing a significant prevalence of 48%. Similarly, Bao et al.’s study revealed that among older patients experiencing early neurological deterioration following acute ischemic stroke, 48.3% were at risk of malnutrition according to GNRI (30). In another study, the GNRI assessment revealed a malnutrition risk of approximately 42.5% (97/228) among older patients with mild traumatic brain injury (31). These results above suggest that brain injury in older patients is often accompanied by malnutrition. This high incidence of malnutrition in the older adults may be attributed to various factors. With advancing age, oral issues such as missing teeth impair chewing ability, impacting food intake and digestion (32). Moreover, older individuals frequently contend with chronic diseases like diabetes and heart disease, potentially increasing metabolic demands and subsequently heightening the risk of malnutrition (33). This study’s findings revealed a significant negative correlation between GNRI and the level of comorbidity, further substantiated that the heightened burden of chronic diseases contributes to the increased risk of malnutrition.

As an infectious disease affecting the nervous system, brain abscesses induce inflammation that escalates the body’s metabolic demands, thereby heightening nutritional requirements (1, 34). This, combined with the adverse effects of infection and pharmacological treatments on appetite, can precipitate a further decline in the nutritional status of patients with brain abscesses (34). In the correlation analysis conducted in this study, the GNRI and CR*p* values upon admission in older patients with brain abscesses were found to be significantly negatively correlated (*p* = 0.006), thereby further indicating the relation between the level of inflammation and the nutritional risk. Furthermore, we observed that patients at nutritional risk underwent more conservative treatment during hospitalization compared to their counterparts without nutritional risk (38.5% vs. 66.7%). This finding implies that the nutritional status of older patients with brain abscess may influence their treatment approach.

In clinical practice, the typical focus on infection control and acute neurological symptom management in brain abscess cases often overshadows the scrutiny of patients’ nutritional status. The findings of this study indicated that older brain abscess patients at higher malnutrition risk exhibit poorer clinical prognoses (*p* < 0.001). A positive correlation emerged between GNRI scores and GOS scores (*p* < 0.001), indicating a nutritional status may be significantly associated with recovery and long-term outcomes in brain abscess patients. Several studies also have demonstrated a correlation between elevated malnutrition risks and adverse clinical outcomes in geriatric patients (31, 35). It can be seen that in the process of diagnosis and treatment of geriatric diseases, the assessment of nutritional status cannot be ignored. However, there is no significant correlation was observed between smoking, alcohol consumption, and either the nutritional status or the GOS in elderly patients with brain abscess in this study. This lack of association is likely due to the standardized, individualized treatment these patients received, which effectively minimized the impact of lifestyle factors on their outcomes (36). The findings emphasize the importance of tailored clinical management in mitigating the potential negative effects of smoking and alcohol use in this patient population.

GNRI has been employed as an independent predictive factor for morbidity and mortality in older hospitalized patients with cancer (37). Research on older colorectal cancer patients revealed that the preoperative GNRI served as an independent prognostic factor for those who underwent curative colorectal resection (38). In another study, the GNRI has been identified as an independent predictor of prognosis and postoperative complications after radical nephroureterectomy for upper urinary tract urothelial carcinoma (39). In addition to its extensive application in forecasting postoperative complications and the long-term prognosis of tumors, the GNRI was also employed in other diseases for its prognostic significance for recuperation and clinical outcomes. A study focused on older patients with mild traumatic brain injury revealed that a higher GNRI was associated with a decreased risk of incomplete recovery at the 6-month mark. Furthermore, the ROC analysis established a GNRI threshold of 97.85 (31). And GNRI was considered a promising tool for predicting mortality outcomes in older patients with moderate to severe traumatic brain injuries (24). These findings indicated that GNRI may have applicability in diseases related to brain injury. In our multivariate regression analysis, we found that lower GNRI values were positively associated with poorer prognosis (i.e., lower GOS score). Moreover, the results of the ROC analysis, which yielded an AUC of 0.903, underscore the significant potential of GNRI in predicting the long-term outcomes for older patients with brain abscess.

Our study offered several strengths. Firstly, as a prospective cohort study, the high standardization of our data collection protocols ensures data quality, while simultaneously reducing selection and recall biases. Secondly, this is the inaugural investigation into the prevalence of nutritional risk among older patients with brain abscesses, providing a novel insight into the clinical implications of GNRI scores. Furthermore, our use of logistic regression analysis has elucidated the relationship between GNRI and the prognostic score for geriatric brain abscesses (GOS), affirming GNRI’s utility in predicting outcomes for older hospitalized patients with brain abscesses. Meanwhile, our study still has certain limitations that should be considered. The study was conducted at a single center, which may limit the generalizability of the findings to other healthcare settings or diverse patient populations. The potential for selection bias and the lack of variability in patient demographics could affect the broader applicability of our results. Although the study utilized a standard nutritional assessment, it did not include specific evaluations for macro or micronutrient deficiencies. This omission may have restricted our understanding of the detailed nutritional profiles of elderly patients with brain abscesses and their impact on treatment efficacy and recovery outcomes. Additionally, reliance on a single prognostic measure, the GOS, may not fully capture the nuanced aspects of patient recovery, suggesting a need for more diverse and sensitive outcome assessment tools in future studies. Therefore, future research should be conducted across multiple centers, incorporate larger patient samples, and integrate additional nutritional assessment methods, such as Dual-energy X-ray Absorptiometry (DXA) or Bioelectrical Impedance Analysis (BIA). To deepen our understanding of the nutritional status of older patients with brain abscess and further explore the impact of nutritional status on treatment and clinical outcomes.

This study on the nutritional status of older patients with brain abscesses revealed that nearly half are at risk of malnutrition according to the GNRI. The GNRI is correlated with the number of comorbidities, aCCI scores, and treatment modalities, underscoring the intricate relationship between nutritional status and the overall health burden in this demographic. Moreover, the study observed that lower GNRI scores in these patients were associated with decreased GOS scores, and found that GNRI is a prognostic indicator of clinical outcomes. Clinically, heightened attention is warranted for patients with GNRI scores below the critical threshold of 97.5, and prompt nutritional interventions should be administered. Consequently, we advocate regular nutritional evaluations for all older patients with brain abscesses, ensuring early intervention with nutritional therapy to preserve their nutritional health and mitigate the risk of adverse outcomes.

## Data availability statement

The original contributions presented in the study are included in the article/Supplementary material, further inquiries can be directed to the corresponding authors.

## Ethics statement

The studies involving humans were approved by Ethics Committee of Huashan Hospital affiliated with Fudan University School of Medicine. The studies were conducted in accordance with the local legislation and institutional requirements. The participants provided their written informed consent to participate in this study.

## Author contributions

XP: Conceptualization, Data curation, Formal analysis, Methodology, Writing – original draft. YZ: Data curation, Formal analysis, Writing – original draft. DJ: Data curation, Methodology, Writing – review & editing. MZ: Data curation, Methodology, Writing – review & editing. JF: Data curation, Writing – review & editing. YN: Supervision, Validation, Writing – review & editing. MT: Supervision, Visualization, Writing – review & editing. SH: Conceptualization, Supervision, Validation, Visualization, Writing – original draft, Writing – review & editing.
