# Supplementary material for: Geriatric nutritional risk index has a prognostic value for recovery outcomes in elderly patients with brain abscess
Source: Front Nutr. 2024 Jul 18;11:1410483. doi: 10.3389/fnut.2024.1410483 (PMC11291442; doi:10.3389/fnut.2024.1410483)
Supplement: Supplementary file 1 [file Table_1.DOCX]

Supplementary Material

**Supplementary Table 1.** **Distribution of GOS scores among elderly patients with brain abscess, categorized by nutritional risk levels according to GNRI**

| **Malnutritional risk according to GNRI** | **Glasgow outcome scale** | | | | |
| --- | --- | --- | --- | --- | --- |
|  | **GOS=1** | **GOS=2** | **GOS=3** | **GOS=4** | **GOS=5** |
| **Major malnutritional risk** | 1 | 3 | 0 | 0 | 0 |
| **Moderate malnutritional risk** | 3 | 2 | 12 | 6 | 0 |
| **Low malnutritional risk** | 2 | 0 | 3 | 9 | 7 |
| **No malnutritional risk** | 5 | 0 | 0 | 1 | 46 |
